# BEST PROSTHESIS FOR UNICOMPARTMENTAL KNEE ARTHROSIS: FIXED OR MOBILE?

**DOI:** 10.1590/1413-785220253301e285052

**Published:** 2025-02-03

**Authors:** Fabrício Luz Cardoso, Deusimar Cristian dos Santos Gomez, Fabrício Roberto Severino, Patrícia Maria Moraes de Barros de Fucs

**Affiliations:** 1 Santa Casa de Misericórdia de São Paulo, Orthopaedic and Traumatology Department, Pavilhão Fernandinho Simonsen, São Paulo, SP, Brazil

**Keywords:** Arthroplasty, Knee Joint, Prostheses and Implants, Weight-Bearing, Artroplastia, Articulação do Joelho, Próteses e Implantes, Suporte de Carga

## Abstract

This study aimed to compare fixed-bearing and mobile-bearing knee unicompartmental arthroplasty implants in adults (in the medial compartment) to determine which is better for each patient and their particularities. The research focused on postoperative assessments with a follow-up of at least a 2-year, examining both quality of life and mid-term functionality in the medium term. A systematic keyword search was executed in the PubMed, EMBASE, and Cochrane databases, employing a filter for randomized clinical trials and without language limitations. The search yielded 113 articles from March 28, 2024, including 83 from PubMed, 12 from EMBASE, and 18 from the Cochrane Library. The study found insufficient evidence to establish the superiority of one prosthetic type over the other regarding post-operative function, pain, complications, revisions, and quality of life after a 2-year follow-up. Literature highlights uncertainties in comparing UKA types due to varied assessment tools. No conclusive evidence favors either type regarding post-op function, pain, complication rates, revisions, or quality of life after 2 years. Urgent need for standardized, long-term, multicenter studies to inform evidence-based clinical practice. ***Level of Evidence I; Systematic review of randomized controlled trials.***

## Resumo

Este estudo teve como objetivo comparar os implantes de artroplastia unicompartimental do joelho, fixos e móveis, em adultos (no compartimento medial), para determinar qual é melhor para cada paciente e suas particularidades. A pesquisa concentrou-se em avaliações pós-operatórias com um acompanhamento de pelo menos 2 anos, examinando tanto a qualidade de vida quanto a funcionalidade a médio prazo. Foi realizada uma busca sistemática de palavras-chave nas bases de dados PubMed, EMBASE e Cochrane, empregando um filtro para ensaios clínicos randomizados, e sem limitações de idioma. A busca resultou em 113 artigos a partir de 28 de março de 2024, incluindo 83 do PubMed, 12 do EMBASE e 18 da biblioteca Cochrane. O estudo encontrou evidências insuficientes para estabelecer a superioridade de um tipo de prótese sobre o outro em termos de função pós-operatória, dor, complicações, revisões e qualidade de vida após um acompanhamento de 2 anos. A literatura destaca incertezas na comparação entre os tipos de artroplastia unicompartimental de joelho devido a ferramentas de avaliação variadas. Não há evidências conclusivas que favoreçam um dos tipos em relação à função pós-operatória, dor, taxas de complicações, revisões ou qualidade de vida após 2 anos. Há uma necessidade urgente de estudos padronizados, de longo prazo e multicêntricos para informar a prática clínica baseada em evidências. **Nível de Evidência I; Revisão sistemática de ensaios clínicos randomizados e controlados.**

## INTRODUCTION

The knee is considered the most complex joint in the human body, defined as a synovial hinge joint.^1^ It consists of three articulations: the medial tibiofemoral joint, the lateral tibiofemoral joint, and the patellofemoral joint.^1^ Its stability relies on the ligaments that connect the femur and tibia, as well as the force and action of the adjacent muscles and their tendons.

Like other joints in the human body, the knee is a strong candidate to undergo degenerative processes, either due to overload or the natural course of aging.^2^ Osteoarthritis or degenerative joint disease (called gonarthrosis when it affects the knee) is clinically characterized by protokinetic pain, claudication, morning stiffness, deformity, and joint enlargement resulting from the interaction between biological and mechanical factors on the articular cartilage, subchondral bone, and synovial fluid.^2^ Radiographically, a reduction in joint space, subchondral sclerosis, bone cysts, and osteophytes are observed.

The condition is considered multifactorial, and among the intrinsic and extrinsic factors that contribute to its development are: age over 60 years (most important), female sex, obesity (most important modifiable factor), genetic predisposition, race, diet, bone metabolism, associated inflammatory or endocrinometabolic comorbidities, activity, occupation, joint/bone, strength, and alignment.^3^ Etiologically, gonarthrosis can be classified as primary or secondary. If there is no well-established known cause, it is called primary, which results from a degenerative process linked to aging; if there is a known cause, it is then referred to as secondary osteoarthritis.

Gonarthrosis can be systematically divided into three types:^4^ I) Inflammatory, resulting from osteoarthritis (a degenerative inflammatory process or due to inflammatory or infectious arthritis, where the subchondral bone lesion is the most relevant); II) Post-Traumatic, which occurs as a consequence of traumas that damage the joint surface, such as fractures and osteochondritis (where the cartilage is most affected); and III) Mechanical, which is a result of axis deviations or joint instabilities, affecting both the cartilage and the subchondral bone.

Although there is a profound understanding of the physiopathology of osteoarthritis, little is still known about the genesis of pain in these patients at the molecular level. Fundamentally, it is known that the possible causes of pain are related to increased intraosseous pressure due to vascular congestion of the subchondral bone, synovitis and inflammation, capsular fibrosis, osteophyte growth, muscle contracture, and weakness.^5^ The maintenance of chronic pain seems to involve both the central and peripheral nervous systems. Initially, hypersensitivity is observed only at the affected site, then mechanisms of central and peripheral sensitization come into play, contributing to the maintenance of painful conditions, independent of the peripheral process that originated the pain, making it refractory.^5^,^6^

Refractory pain to clinical treatment, non-pharmacological measures (intra-articular injection of hyaluronic acid, shockwave therapy, physiotherapy, among others), or surgical procedures (such as knee arthroscopy, synovectomies, osteotomies, among others) are the main factors that lead to the indication for knee arthroplasty.^7^ Similar to other joints, the knee can also develop a form of osteoarthritis resulting from the progression of muscular imbalance^2^, which stimulates the development of a mechanical type of osteoarthritis with well-defined characteristics. Specifically in the knee, this condition affects the medial compartment, promoting a varus deformity^4^, and in the absence of treatment, the degenerative process evolves progressively.

Arthroplasties aim to relieve pain, correct deformities, improve joint motion, and enhance quality of life^7^. Unicompartmental knee arthroplasty (UKA) has been performed since the early 1970s,^8^ with advancements in implant design and surgical techniques in recent decades improving outcomes. UKA is indicated for localized knee degeneration, maintaining ACL integrity and limb alignment, and requiring good bone quality. It benefits patients with low activity levels or localized osteoarthritis, potentially offering faster recovery compared to total knee arthroplasty (TKA). However, UKA suitability should be carefully assessed by a specialized orthopedic surgeon, considering individual patient characteristics and needs.

In UKA, distinguishing between types is vital for selecting the appropriate prosthetic device based on patient needs and anatomy.^7^,^9^ Key factors include tibial component fixation (cemented versus uncemented), component material (fully poly versus metallic), and replacement location (medial versus lateral). UKA implants are categorized as fixed-bearing (FB), where a polyethylene structure is fixed between femoral and tibial components, and mobile-bearing (MB), which allows anterior and posterior mobility of the polyethylene, unlike MB implants in total knee replacements, which also permit rotational movements^7^,^9^

In UKA, the choice between FB and MB involves considerations of distinct advantages and disadvantages.^7^,^9^ As of the present moment, the literature has not clearly defined the superiority of one implant type over the other when compared (FB vs. MB). Both have advantages, disadvantages, and indications related to intrinsic and extrinsic factors of the patient are crucial in choosing the best prosthesis type for the treatment of knee conditions. FB offers stability and simplicity of design, facilitating surgery and reducing the risk of dislocation. Additionally, wear tends to be more uniform, extending the prosthesis lifespan. However, it may limit range of motion and increase stress on the joint, potentially contributing to adjacent bone wear. On the other hand, MB allows for greater range of motion and more natural load distribution, reducing stress on the joint and potentially minimizing adjacent bone wear. However, surgery may be more complex due to the need to ensure adequate stability of the mobile implant, and there is a slightly increased risk of dislocation.

The objective of this study is to determine the most suitable prosthesis type for individual patients by comparing their indications in the adult population. Post-operative evaluations of patients with a minimum follow-up of 2 years were conducted, focusing on aspects such as quality of life and medium-term post-operative function. The choice of a 2-year follow-up period is considered medium-term as it allows for the assessment of both short-term recovery and early outcomes as well as the beginning of potential long-term effects.

## MATERIALS AND METHODS

This systematic review (Level of evidence: 1) was submitted in its inception to the PROSPERO® platform^10^ under the registration number CRD42022383120 with the aim of minimizing the risk of publication bias and the duplication of reviews to address the same clinical question.

A literature search was conducted in the search engines of the following databases: PubMed, EMBASE, and Cochrane library, using the following keywords: “Fixed AND Mobile AND knee arthroplasty, unicompartmental.” The search was refined to include only randomized clinical trials without language restrictions, up March 28, 2024.

The inclusion criteria were as follows: (I) Full articles of randomized clinical trials comparing the use of FB with MB unicompartmental knee arthroplasty (UKA) in the treatment of unicompartmental knee osteoarthritis; (II) Studies that evaluated patients with a follow-up of at least two years (2) post-operatively allowing for shorter post-operative assessments as long as they were compared with an evaluation of at least two years of follow-up. The exclusion criteria were: I) Duplicated articles, where the abstract is published in one journal and the full article in another (opting for the full article and excluding the abstract) and; II) Articles that appeared in more than one database (using only one of the articles in the quantification and review). After organized the articles following the PRISMA® flowchart.^11^

In order to use data that support evidence-based medicine, the PICO strategy^12^ represents an acronym for Patient, Intervention, Comparison, and Outcomes. These four components are fundamental elements for formulating a good research question and constructing the clinical question for literature search for evidence.^12^ The components are specified as follows:

Patient: Adult population, regardless of race, sex, and health history, with unicompartmental knee osteoarthritis.

Intervention: Surgical treatment of unicompartmental knee osteoarthritis with MB or FB partial knee prosthesis.

Comparison: Clinical outcomes and complications of unicompartmental (partial) knee prosthesis between the groups: MB vs. FB, using evaluation tools.

Outcome: Pain, knee joint function, quality of life, post-operative complications, and revisions, considering a minimum follow-up of 2 years.

The data treatment of Table generated after applying the PICO tool^12^ was conducted using a double-check technique by two authors. Each author’s input was reviewed, and contributions and additions were made by the other author, aiming to avoid data selection bias and include the main aspects covered in each of the studies used as the basis for the systematic review.

Furthermore, the ROBIS® tool^13^ (Risk of Bias in Systematic Reviews) was used: an instrument applied to assess the risk of bias in systematic reviews. This tool was designed to evaluate bias risk with questions related to interventions, etiology, diagnosis, and prognosis. Therefore, it is considered an appropriate choice of tool for the scope of this systematic review work: evidence-based medicine in the field of orthopedics and traumatology, specifically concerning orthopedic prostheses applied to knee surgery.

In the initial step of assessing relevance, it was determined that the subject discussed in the review is aligned with the research question intended to be addressed. The second stage involved the assessment of four domains to cover the main review processes: 1) study eligibility criteria; 2) identification and selection; 3) data collection and evaluation of studies; and 4) synthesis and results. The study answered all questions leaving no doubts about its pre-established methodology and registered on the PROSPERO® platform.^10^ In the third and final stage, the assessment focused on evaluating the risk of bias. The first question in this phase revealed that the interpretation of the findings encompassed all potential risks and no biases were identified. Additionally, this phase comprised three questions related to the interpretation of the review findings. These questions demonstrated that the conclusions were grounded on the presented evidence, the relevance of the included studies was taken into account, and the authors refrained from solely emphasizing results based on statistical significance. Such considerations are vital for properly interpreting the findings of a review, as they are potential areas where biases could have been introduced into the study.

Using all the tools mentioned above, it is possible to ensure the reproducibility of the study.

## RESULTS

**Figure 1:**
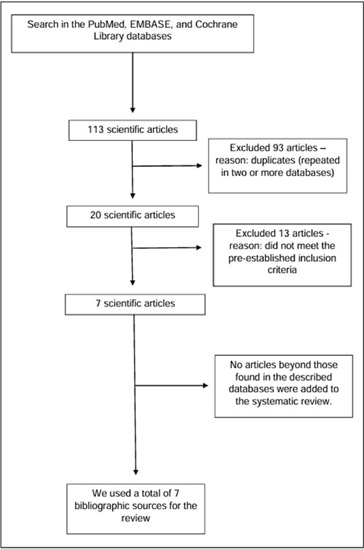
Flowchart designed for the search and selection of studies for the review.

A total of eighty-three (83) articles were found in PubMed, twelve (12) articles in EMBASE, and eighteen (18) search results in the Cochrane library, associated with the described themes up to March 28, 2024, totaling one hundred and thirteen (113) search results. After analyzing the articles following the PRISMA® flowchart^11^ (Figure 1), there were seven (7) remaining references.

The references were manually reviewed and arranged in chronological order of publication. Seven (7) articles were included, all written in English and published between 2003 and 2024 (Table 1). The total number of patients evaluated in the studies^14^,^15^,^16^,^17^,^18^,^19^,^20^ that composed this systematic review was 525, with 538 knees operated and evaluated with a minimum follow-up of 2 years. The overall mean age of the studies was 68,67 years.

**Table 1:** Material used in the review.

Order	Authors	Journal [Database]	Year	Authors’ country	Language
**Artigos das bases de dados**					
1	Gleeson RE; Evans R; Ackroyd CE; Webb J; Newman JH^14^	The Knee [Pubmed; Cochrane Library]	2003	United Kingdom (England)	English
2	Confalonieri N; Manzotti A; Pullen C^15^	The Knee [Pubmed; EMBASE; Cochrane Library]	2004	Italy Australia	English
3	Li MG; Yao F; Joss B; Ioppolo J; Nivbrant B; Wood D^16^	The Knee [Pubmed; EMBASE; Cochrane Library]	2006	Australia	English
4	Gilmour A; MacLean AD; Rowe PJ; Banger MS; Donnelly I; Jones BG; et al^17^	The Journal of Arthroplasty [Pubmed; EMBASE; Cochrane Library]	2018	United Kingdom (Scotland)	English
5	Koppens D; Rytter S; Munk S; Dalsgaard J; Sørensen OG; Hansen TB; et al.^18^	Acta Orthopaedica [Pubmed; EMBASE; Cochrane Library]	2019	Denmark	English
6	Wu L; Mayr HO; Zhang X; Huang Y; Chen Y; Li Y^19^	Orthopaedic Surgery [Pubmed; EMBASE; Cochrane Library]	2022	China Germany	English
7	D’Ambrosi, R.; Valli, F. ; Nuara, A.; Mariani, I.; Di Feo, F. ; Ursino, N.; Formica, M.; Mangiavini, L.; Hantes, M.; Migliorini, F.^20^	European Journal of Orthopaedic Surgery & Traumatology	2023	Italy Greece Germany	English

Table 2 displays information derived from the PICO data treatment strategy,^12^ following a meticulous data processing procedure executed by two authors employing a double-check technique. After using the ROBIS tool,^13^ no bias was identified in our study.

**Table 2:** Detailed data of the references.

Order	Tipe of Study	Pacient Population	Intervention	Comparison	Outcomes
1^14^	Randomized Clinical Trial	Period and recruited population: Between January 1999 and December 2001, 91 patients (104 knees) were recruited for the study. One patient had different arthroplasties implanted in each knee. Preoperative diagnoses and previous surgical procedures: The preoperative diagnosis was primary osteoarthritis in all cases, except for two (one case of osteonecrosis and one of rheumatoid arthritis). Previous surgeries included arthroscopic procedures, medial meniscectomies, and, in one case, anterior cruciate ligament (ACL) reconstruction. Excluded population: Patients with prior tibial plateau fracture or knee osteotomy were excluded. Chondrocalcinosis was not considered a contraindication, and no patients were excluded based on excess weight. Division of the comparison groups and characteristics of each group (sex, number of patients, mean age, mean body weight): St. George Group (Fixed-Bearing): 57 knees in 49 patients; Mean age: 66.7 years; 29 males and 20 females; 26 right knees, 31 left knees; Mean body weight: 83.0 kg Oxford Group (Mobile-Bearing): 47 knees in 43 patients; Mean age: 64.7 years; 26 females, 17 males; 25 right knees, 22 left knees; Mean body weight: 77.7 kg	Types of prostheses used: Mobile-Bearing Unicompartmental Knee Prosthesis (Oxford) and the Fixed-Bearing Unicompartmental Knee Prosthesis (St. George). Clinical indications for surgery: The indications for unicompartmental knee prosthesis were: Incapacitating knee pain with medial compartmental disease; Intact anterior cruciate ligament (ACL) and collateral ligaments; Fixed flexion deformity less than 108 degrees; Minimal subluxation; and correctable varus deformity less than 108 degrees.	Comparison proposed by the work: The complications and clinical outcomes of the St. Georg Sled, a fixed-bearing unicompartmental knee prosthesis, with the mobile-bearing Oxford unicompartmental knee prosthesis over a two-year postoperative period. Assessment tools and time of evaluation (preoperative, postoperative, or both): The Bristol Knee Score (BKS) and Oxford Knee Score were used to assess knee function preoperatively, at 8 months, and 2 years postoperatively. Preoperative weight, range of motion, and knee scores for each group were the variables studied. No patients were lost in follow-up, and 88 out of 91 patients attended the 2-year postoperative evaluation.	Função: Bristol Knee Score and Oxford Knee Score: At the 2-year follow-up, both socres showed better outcomes for the St. Georg Sled Group (fixed-bearing). There were also more excellent and good results in the St. Georg Sled Group. However, there was no significant difference compared to the Oxford Group (mobile-bearing). Mean 2 years after post-op Bristol knee score FB / MB 89 / 84.1 Mean 2 years after post-op Oxford score FB / MB 36.5 / 33.4 Mean total pain score (component of Bristol knee score, max=40) FB / MB 34.9 / 30.7 Mean total function score (component of Bristol knee score, max=27) FB / MB 23 / 22 Mean of flexion post-op (range of motion) FB / MB 121.68 / 118.68 Pain: The pain component of the Bristol Knee Score was significantly better for the St. Georg Sled Group, fixed-bearing (p-value = 0.013). Postoperative complications and revisions: In the Oxford Group (mobile-bearing), three patients experienced bearing dislocation, and four patients required revisions with an average revision time of 3 years. In the St. Georg Sled Group (fixed-bearing), three patients required revisions with an average revision time of 3.4 years. Comparative conclusions on quality of life: These results demonstrate that, in the short term, the Oxford mobile-bearing prosthesis has a higher reoperation rate, while the fixed-bearing St. George sled prosthesis achieves better pain relief. The functional scores of both groups were similar.
2^15^	Randomized Clinical Trial	Period and recruited population: Between February 1996 and December 1997, 40 patients who underwent medial unicompartmental knee arthroplasty were recruited and randomly divided into two groups. Preoperative diagnoses and previous surgical procedures: Primary osteoarthritis Excluded population: Division of the comparison groups and characteristics of each group (sex, number of patients, mean age, mean body weight): Group A - 20 knees in 20 patients; mean age 69.5 years; 8 males and 12 females; 11 left knees; 9 right knees. Group B - 20 knees in 20 patients; mean age 71 years; 11 males and 9 females; 8 left knees; 12 right knees.	Types of prostheses used: Group A -Allegretto, Centerpulse, Baar, Switzerland (Fixed-bearing) and Group B - - AMC-Unicondylar-Knie-Prothese, Alphanorm, Quiershied, Alemanha (Mobile bearing) Clinical indications for surgery:	Comparison proposed by the work: Pre- and postoperative follow-up with clinical and statistical evaluations using scores, with an average postoperative follow-up period of 5.7 years. Assessment tools and time of evaluation (preoperative, postoperative, or both): The patients were evaluated preoperatively by two independent orthopedists who were not involved in the surgical procedure and were blinded to the type of prosthesis implanted. Additionally, the Knee Society scoring instrument, the G.I.U.M. (Unicompartmental Knee Prosthesis Outcome Score developed by the Italian Orthopaedic UKR’s Users Group), and the Functionality Score were used for comparison..	Function: Both in the preoperative and at the last follow-up consultation, no statistically significant differences were detected between the two groups according to the GIUM Score, the Knee Society Score, or the functional evaluation of the patients. Group A: pre / post Knee Society: 44,6 / 87.5 Functional: 48.7 / 76.3 GIUM: 51.3 / 73.8 Group B: pre / post Knee Society: 48.3 / 88.05 Functional: 48.7 / 77.0 GIUM: 52.4 / 75.5 Pain: Postoperative complications and revisions: After 18 months, one patient in Group A underwent a revision of the prosthesis due to persistent pain in the tibial component region, with only partial reduction of the pain complaint after the procedure. In one patient from Group B, an intraoperative medial tibial plateau fracture occurred, which was treated with screw fixation before implantation of the tibial component and did not affect the final outcome or postoperative follow-up. One patient with the fixed component had a TVP, but the condition was treated without complications. There were no cases of superficial or deep infections. Comparative conclusions on quality of life: These results demonstrated that despite more extensive usage, it was not possible to detect advantages of the mobile-bearing prosthesis over the fixed-bearing prosthesis in terms of clinical performance and longevity.
3^16^	Randomized Clinical Trial	Period and recruited population: Between May 2001 and June 2003, 56 knees in 48 patients, 34 males, 14 females, with a mean age of 72 years and a diagnosis of osteoarthritis were randomly assigned to two groups for knee arthroplasty. Preoperative diagnoses and previous surgical procedures: Non-inflammatory osteoarthritis of the medial compartment and/or mechanical deformity. Excluded population: Division of the comparison groups and characteristics of each group (sex, number of patients, mean age, mean body weight): Fixed-Bearing Group (Miller/Galante, Zimmer, Warsaw, USA): 28 knees: 19 males, 9 females; Mean age: 70 years; Mean BMI: 27.6 Mobile-Bearing Group (Oxford, Biomet, UK): 28 knees: 20 males, 8 females; Mean age: 74 years; Mean BMI: 26.5 Eight patients received bilateral implants (always with one knee receiving an Oxford implant and the other receiving a Miller/Galante implant).	Types of prostheses used: Fixed bearing (Miller/Galante, Zimmer, Warsaw, USA Mobile bearing (Oxford, Biomet, UK) Clinical indications for surgery: Non-inflammatory osteoarthritis of the medial compartment, mechanical axial deformity <10° varus or 5° valgus; intact ACL without medial-lateral subluxation; Flexion contracture <15°; Body weight <90 kg.	Comparison proposed by the work: Comparison of fixed-bearing and mobile-bearing knee prostheses, with a focus on knee kinematics, tibial component radiolucency, and clinical follow-up over a period of 2 years. Assessment tools and time of evaluation (preoperative, postoperative, or both): The comparison was conducted based on the following three criteria: Kinematic: 1) Internal rotation of the tibia relative to the femur. 2) Anterior-posterior translation of the medial femoral condyle. 3) Anterior-posterior translation of the contact point. 4) Movement of the mobile-bearing. Radiographic: Comparison of postoperative radiographs immediately after the procedure with those taken after 2 years. Alignment assessed through the Hip-Knee-Ankle (HKA) angle. Radiolucency at the bone-implant interface. Progression of osteoarthritis in the patellofemoral joint and the lateral component. Positioning and alignment of the tibial and femoral components. Clinical: Independent observers evaluated preoperatively and annually during follow-up using scores such as Knee Society Scores, WOMAC, and SF-36	Function: The mobile-bearing prosthesis showed a closer approximation to the normal knee kinematics, with greater and more consistent tibial internal rotation, a more stationary medial femoral condyle, and rollback of the lateral femoral condyle. It also presented a lower incidence of radiolucency (with increased radiolucency being a possible sign that the fixation quality may be compromised in this group, though longer follow-up time is necessary to confirm this fact). Fixed / Mobile Range of motion: 110 (85–140) / 112 (90–135) Knee score: 91 / 89 Function score 84 / 85 Pain: After 2 years Fixed / Mobile 46 / 44 SF-36 score Preoperative physical: 27 / 29 2 years, physical 37 / 40 2 years, mental 52 / 50 Womac score Pre 46 / 54 2 years 74 / 79 Postoperative complications and revisions: Two patients with a mobile-bearing prosthesis require prosthesis revision before two years: one due to infection and the other due to aseptic loosening of the tibial component. Two patients with 3 prostheses (one mobile-bearing and two fixed-bearing) died from unrelated causes before two years. They were excluded from the final comparison but included in the initial study data. Comparative conclusions on quality of life: Both SF-36, Womac, and Knee Society scores improved during the two-year follow-up period with no significant differences between the two groups.
4^17^	Randomized Clinical Trial	Period and recruited population: Between October 2010 and December 2012, a total of 139 participants were recruited. Preoperative diagnoses and previous surgical procedures: Excluded population: Patients with ligament insufficiency, inflammatory arthritis, deformity requiring augmentation, neuromotor diseases, pathologies of the feet, ankles, hips, or contralateral knee causing significant pain or gait alteration, as well as those requiring total knee replacement. Division of the comparison groups and characteristics of each group (sex, number of patients, mean age, mean body weight): Dividided into two groups: Fixed-Bearing Group: 69 initial participants; 64 underwent the procedure; 58 were followed for 2 years; 32 males and 26 females; Mean age of 61.8 years Mobile-Bearing Group: 70 initial participants; 65 underwent the procedure; One patient crossed over from the other group; 54 were followed for 2 years; 28 males and 26 females; Mean age of 62.6 years; Two patients were not followed due to the need for total knee replacement revision.	Types of prostheses used: Fixed-Bearing: Surgical technique assisted by a robotic arm using the RESTORIS MCK (MAKO Surgical Corp, Fort Lauderdale, FL) with the MAKO Robotic-Arm Interactive Orthopedic system. Mobile-Bearing: Conventional surgical technique using the Oxford Phase 3 (Biomet, Warsaw, IN) prosthesis. Clinical indications for surgery: Osteoarthritis of the medial compartment of the knee requiring surgery	Comparison proposed by the work: Unicompartmental knee prosthesis with robotic-assisted surgical technique and conventional prosthetic technique, performed by one of the three surgeon authors with at least 5 years of experience in independent practice. Assessment tools and time of evaluation (preoperative, postoperative, or both): For the comparison, several scores were used, including OKS (Oxford Knee Score), AKSS (American Knee Society Score), Forgotten Joint Score (FJS), Pain Catastrophizing Scale, Pain Visual Analog Scale, Stiffness Visual Analog Scale (SVAS), patient satisfaction, range of motion (ROM), and University of California Los Angeles (UCLA) Activity Scale. Complications and revisions over the 2-year period were also taken into account. Data collection was performed by an associate/research nurse who was blinded to the group data at the investigative hospital.	Function: The SVAS was significantly higher in the manual group, while the ROM was greater in the robotic-assisted surgery group, and both remained consistent after 2 years of follow-up. Pain: After 2 years Mobile / Fixed Pre: 55.1 / 52.7 After 2 years: 5.0 / 3.0 Postoperative complications and revisions: From the mobile-bearing group, two patients were lost to follow-up due to the need for total prosthesis revision. Regarding the evaluation of survival differences (100% in the assisted group and 96.3% in the conventional group), long-term follow-up is necessary. Comparative conclusions on quality of life: At two years, no significant differences were detected by the study’s analysis tools. However, in the subgroup of patients with a preoperative University of California Los Angeles Activity Scale value >5, a higher postoperative mean of the Oxford Knee Score was observed after two years, indicating a possible greater benefit of robotic-assisted surgery for more active patients. Nevertheless, longer follow-up time is required to draw conclusive results.
5^18^	Randomized Clinical Trial	Period and recruited population: Between January 2014 and November 2015, a total of 62 patients were followed through stereometric analysis by radiography.. Preoperative diagnoses and previous surgical procedures: Excluded population: Patients with inflammatory arthritis, contralateral knee prosthesis, disseminated malignancy, severe systemic disease, female patients of childbearing age, and patients unable to provide written consent. Division of the comparison groups and characteristics of each group (sex, number of patients, mean age, mean body weight): Mobile-Bearing Group (Oxford UKA): 33 patients, of which 2 did not undergo the procedure (due to no LCA); Mean age of 64 years; 16 males and 17 females; Mean BMI of 29 Fixed-Bearing Group (Sigma UKA): 32 patients, of which 1 did not undergo the procedure and 1 was excluded due to infection 5 weeks after the procedure; Mean age of 61 years; 17 males and 15 females; Mean BMI of 28.	Types of prostheses used: Mobile bearing (Oxford UKA) Fixed bearing (Sigma UKA) Clinical indications for surgery: Patients above 18 years of age eligible for unicompartmental knee prosthesis according to the criteria established by Murray et al. (1998) and DePuy International (2009).	Comparison proposed by the work: The unicompartmental knee prostheses implanted by two experienced orthopedic surgeons using minimally invasive techniques. During the surgery, tantalum beads measuring 4-6 mm were implanted in the femoral and tibial periprosthetic regions for subsequent RSA. Assessment tools and time of evaluation (preoperative, postoperative, or both): Radiostereometric analysis (RSA) was performed on postoperative day one and subsequently at 4, 12, and 24 months. All RSA data were analyzed using the same system, and patients with fewer than 3 visible markers were excluded from the analysis. In addition to RSA, the study also utilized the Oxford Knee Score and a general health questionnaire (RAND-36) to assess overall health. Furthermore, the strength of the thigh extensors was evaluated, with both lower limbs being tested preoperatively and again after 24 months.	Function: Oxford Knee Score MB / FB Pre: 26 / 28 4 months: 38 / 37 12 months: 42 / 41 24 months: 40 / 41 Pain: Após 2 anos RAND-36 MB / FB Pre: 65 (44) / 72 (38) 4 months: 77 (38) / 87 (32) 12 months: 85 (36) / 81 (34) 24 months: 87 (32) / 91 (23) Postoperative complications and revisions: Mobile-bearing: 02 patients did not undergo the procedure (absence of ACL). Fixed-bearing: 01 patient did not undergo the procedure, and 01 patient was excluded due to infection 5 weeks after the procedure. Comparative conclusions on quality of life: No statistically significant or clinically relevant differences were observed. There was recovery of function and strength of the extensor muscles, with no noted changes between the limbs after 24 months of follow-up, and good fixation was observed during the same period.
6^19^	Randomized Clinical Trial	Period and recruited population: From September 2015 to February 2017, a prospective, randomized, parallel, and single-center study was conducted with 180 patients Preoperative diagnoses and previous surgical procedures: Medial compartmental knee osteoarthritis was performed Excluded population: (i) Patients with lateral compartment knee osteoarthritis, knee arthroplasty in the contralateral knee, inflammatory arthritis, and disseminated malignancies such as AIDS, syphilis, and hepatitis B; (ii) Severe systemic diseases, such as rheumatoid arthritis and malignancies; (iii) Revisional arthroplasty and postinfection cases; (iv) Female patients of childbearing age; (v) Patients unable to provide written informed consent. Division of the comparison groups and characteristics of each group (sex, number of patients, mean age, mean body weight): 78 men and 102 women, with an overall mean age of 63.3 ± 6.9 years, divided as follows: MB: 60 patients, mean age of 63 years, mean BMI of 24 FB: 60 patients, mean age of 63 years, mean BMI of 24 TKA: 60 patients (data not used in this review as it concerns total knee replacement).	Types of prostheses used: Unicompartmental knee prosthesis with fixed or mobile bearing, or total knee arthroplasty. Mobile: Oxford phase 3 MB UKA Fixed: Link FB UKA Total: Depuy Sigma PFC PFC TKA Clinical indications for surgery: The inclusion criteria were: (i) Patients aged between 50 and 80 years at the time of recruitment, with clinical and radiographic evidence (including anteroposterior and lateral knee radiographs and knee computed tomography [CT]) of non-lateral compartment knee osteoarthritis, with Kellgren-Lawrence X-ray classification levels 2-4. (ii) Competent and willing to participate in the study. (iii) Absence of signs of any severe neurological disorders. (iv) Provided informed consent for the treatment and testing program.	Comparison proposed by the work: A similar perioperative management and fast-track surgery program were implemented for all patients. Knee scores at the 3-year follow-up after the operation, as well as the clinical outcomes of these three patient groups, were recorded, investigated, and compared. Various parameters were also recorded, investigated, and compared, including operative time, intraoperative bleeding, time to the first walk without crutches, independent stair ascent and descent after the operation, postoperative complications, and a series of knee scores. Assessment tools and time of evaluation (preoperative, postoperative, or both): The following scores were used: Hospital for Special Surgery Knee Score (HSS) Western Ontario and McMaster Universities Index (WOMAC) Pontuação da Knee Society (KSS) Visual Analog Scale (VAS) Oxford Knee Score (OKS) Maximum knee flexion angle Forgotten Joint Score (FJS) Follow up exceeding 36 months	Function: Overall, there was no significant difference in all knee scores and maximum knee flexion angles between the MB UKA and FB UKA groups. Pain: After 3 years WOMAC Pre: MB - 47,5 / FB - 47.5 3 years: 91 VAS Pre: MB - 9.0 / FB - 9.0 3 years: 1.0 Postoperative complications and revisions: There was one case of the original dislocation of the bearing in the MB UKA group. In the FB UKA group, one patient had femoral component dislocation caused by a fall injury, and another patient lost their life in a car accident. Comparative conclusions on quality of life: This study indicates that there are no significant differences, with similar Knee Scores between patients with MB and FB. A randomized control study using radiostereometric analysis at the 2-year follow-up showed that both groups have good fixation of the tibial components and both demonstrate good clinical progress. The groups also showed significant improvement in pain and function, evolving significantly up to 12 months postoperatively.
7^20^	Randomized Clinical Trial	Period and recruited population: A total of 54 patients were recruited during the period from September 2015 to December 2019. Preoperative diagnoses and previous surgical procedures: Patients with idiopathic or secondary osteoarthritis of the medial femoral compartment of the knee. Excluded population: The exclusion criteria were: (1) age < 80 years; (2) revi sion arthroplasty; (3) previous surgery of the affected knee (except meniscectomy); (3) uncontrolled systemic disease; (5) patient unable to understand the nature of the present study Division of the comparison groups and characteristics of each group (sex, number of patients, mean age, mean body weight): FB PKA Persona Partial Knee (PPK) group: 25 patients; mean age 82.3 ± 2.0; 23 women and 2 men. MB PKA Oxford: 29 patients; mean age 81.9 ± 1.0; 25 women and 4 men.	Types of prostheses used: The first group received FB PKA Persona Partial Knee (PPK) ® (Zimmer Biomet, Warsaw, Indiana, USA); The second received MB PKA Oxford with Microplasty instrumentation (Zimmer Biomet, Warsaw, Indiana, USA). Clinical indications for surgery: Patients with idiopathic or secondary osteoarthritis of the medial femoral compartment of the knee; Varus or valgus deformity < 3°; Knee flexion > 100°; Flexion contracture < 10°; Integrity of cruciate and collateral ligaments.	Comparison proposed by the work: The patients were assessed at T0 (preoperative), T1 (1 year post-surgery), and T2 (3 years post-surgery).The hypothesis of the current study was that MB implants would perform better than FB implants in PKA in octogenarians. Assessment tools and time of evaluation (preoperative, postoperative, or both): Using visual analogue scale (VAS), Knee Society Score (KSS), and Oxford Knee Score (OKS). Additionally, data on implant survival and range of motion (ROM) were collected. Furthermore, the following radiographic parameters were measured: Varus/valgus of the femoral component; Varus/valgus of the tibial component; Anteroposterior slope.	Function: No difference between FB and MB in KSS, and OKS. KSS Pre: FB 37.3 ± 8.2 / MB 37.1 ± 9.6 (p=0.9) 3 year: FB 90.8 ± 5.5 / MB 90.9 ± 4.9 (p=0.9) OKS Pre: FB 21.8 ± 3.7 / MB 21.2 ± 3.7 (p=0,05) 3 year: FB 43.8 ± 1.7 / MB 43.8 ± 1.7 Pain: No difference between FB and MB in VAS. VAS Pre: FB 7.4 ± 1.2 / MB 7.4 ± 1.5 (p=0.9) 3 year: FB 1.4 ± 0.9 / MB 1.5 ± 0.9 (p=0.8) Postoperative complications and revisions: At last follow-up (3 years), FB group reported three failures caused by aseptic loosening. Four failures were observed in the MB cohort: two for bearing dislocation and two for aseptic loosening. The Kaplan–Meier Curve found no differences in implant survivorship. Comparative conclusions on quality of life: According to the main findings of the present clinical trial, MB implants performed similar to FB in PKA in octogenarians. The FB group demonstrated shorted surgical time. No difference was found in patient reported outcome measures, ROM, implant positioning, and survivorship.

## DISCUSSION

Knee unicompartmental osteoarthritis is a relatively common condition;^21^ however, determining the best type of surgical treatment or the optimal prosthetic type remains controversial. The UKA, with its promise of being a less invasive alternative to total knee arthroplasty (TKA) and proximal tibial and distal femoral osteotomies in suitable patients, continues to attract surgeons and patients. The usual options for partial prostheses are the MB and FB options.

Arliani et al.,^22^ in their study conducted ten years ago on surgical indications, interviewed 113 knee specialists. The majority of participants (89.3%) considered patients under the age of 65 as ideal candidates for UKA, with 95.6% indicating high tibial osteotomy and 74.3% recommending UKA for young patients (<55 years) with high physical demands. Currently, Belsey et al.^23^ suggest in their systematic review that the ideal patient for osteotomy would have compartmental osteoarthritis, tibial deformity, knee mobility greater than 120 degrees, be below 60 years of age, and have a body mass index (BMI) less than 30 kg/m. Conversely, the ideal candidates for UKA would be patients with degeneration, mainly compartmental, but aged over 60 years, with deformity less than 15 degrees, and in both groups, no significant instability should be present.^23^,^24^,^25^ The overall mean age of the studies was 68,67 years (indicating that the elderly population is responsible for the majority of procedures) and the most frequent was primary osteoarthritis in all studies.^14^,^15^,^16^,^17^,^18^,^19^,^20^

However, the consideration that candidates for UKA should be older and less active has been questioned in the literature, as described by Salman et al.^26^ in their meta-analysis of 6130 knees, which concluded that young age was not associated with a higher rate of revisions or lower functional scores, and age alone is not a contraindication for UKA. Regarding the mean BMI of patients undergoing partial knee prostheses in the studies that utilized this index, it was 26.5 (overweight or pre-obese), which differs from the study by Camanho et al.,^8^ which was conducted 15 years ago when obesity was considered an absolute contraindication for UKA due to limitations in the surgical technique.

Among the preoperative diagnoses, the most frequent was primary osteoarthritis in all studies.^14^,^15^,^16^,^17^,^18^,^19^,^20^ This fact demonstrates that osteoarthritis of inflammatory etiology or degenerative nature without inflammation predominates over post-traumatic and mechanical causes.

Function: Despite the variety of tools used by different studies to compare the function of UKA, only one of the studies^14^ showed a difference in results, favoring the FB, but also highlighting the technical difficulty of using the Oxford prosthesis (MB). This isolated result in favor of the FB is contradicted by the meta-analysis conducted by Migliorini et al.^27^, where 4696 patients were assessed and the authors reported not being able to identify the superiority of one implant type over the other, with no differences found in the range of motion (p = 0.05), Knee Scoring System (p = 0.9), function subscale (p = 0.2), and Oxford Knee Score (p = 0.4).

Pain after 2 years: Only one study^14^ showed a slightly lower pain component in the Bristol score in favor of the FB prosthesis (St. Georg Sled). The meta-analysis by Zhang et al. assessed 17 studies involving 2612 knees (with a mean follow-up time ranging from 7 months to 17.2 years) and no significant differences were observed in clinical and radiological outcomes between MB and FB prostheses.

Postoperative complications and revisions: Some cases of bearing dislocation were recorded in the MB groups,^14^,^15^,^16^,^17^,^18^,^19^,^20^ but the rates of prosthesis revision did not show significant differences between the groups. Other postoperative complications that were not explored in this study, such as postoperative infection, also did not have statistically significant values to distinguish between the groups. This finding aligns with the results found by Migliorini et al.,^27^ who described no difference in revision rate (p = 0.2), aseptic loosening (p = 0.9), deep infections (p = 0.99), fractures (p = 0.6), and additional extension of osteoarthritis to the contralateral joint compartment (p = 0.2) between the two prosthesis types in the 4696 patients analyzed in their meta-analysis. The data is also in line with the systematic review by Ko et al.^28^, which evaluated the overall reoperation rate per hundred component years in 1,019 knees from 887 patients. This rate was similar between mobile bearings (1.392) and fixed bearings (1.377).

Comparative conclusions on Quality of Life: In this aspect, no differences were detected, with significant improvement observed for both groups. Regarding sports activities after UKA, Arliani et al.^22^ reported that the most authorized sports by physicians were swimming (96.5%) and tennis (51.3%), while football was disallowed in the postoperative period by all participating surgeons. There are no studies in the literature demonstrating differences in the return to sports after surgery in patients undergoing UKA with FB or MB prostheses. However, the study by Belsey et al.^23^ compared UKA with high tibial osteotomy and concluded that both techniques allow a return to sports activity at a similar or even higher level than the preoperative period. Patients undergoing osteotomies usually exhibit a higher level of physical activity in the pre and postoperative periods. Surprisingly, patients with UKA showed a greater increase in physical activity in the postoperative period compared to what they practiced preoperatively.

The study recognizes several limitations that should be considered when interpreting its findings. Challenges include the difficulty in reaching definitive conclusions due to the lack of standardization in assessment tools, introducing variability that may impact outcome precision. Additionally, the limited availability of relevant literature poses a challenge, with few studies demonstrating the superiority of one model over another in postoperative aspects. The inclusion criteria further narrowed the selection to a small quantity of articles (7 articles), emphasizing the need for caution in generalizing findings to a broader context.

## CONCLUSION

Based on what is described in the literature, there are still numerous questions regarding the comparison of the two types of UKA. The major challenge in reaching conclusions is the standardization of assessment tools, as different variables can be observed depending on the tool used. What is known so far is that there are not enough studies to prove the superiority of one prosthesis type over the other concerning postoperative function, pain after a 2-year follow-up, complication rates, postoperative revisions, and quality of life. Prospective and multicenter long-term studies with standardized methodologies need to be conducted to clarify the doubts that still surround the scientific community to provide evidence-based clinical practice.
